# Determination of Predominant Organic Acid Components in *Malus* Species: Correlation with Apple Domestication

**DOI:** 10.3390/metabo8040074

**Published:** 2018-10-31

**Authors:** Baiquan Ma, Yangyang Yuan, Meng Gao, Cuiying Li, Collins Ogutu, Mingjun Li, Fengwang Ma

**Affiliations:** 1 State Key Laboratory of Crop Stress Biology for Arid Areas, Shaanxi Key Laboratory of Apple, College of Horticulture, Northwest A&F University, Yangling 712100, China; bqma87@nwsuaf.edu.cn (B.M.); yy.yuan@nwsuaf.edu.cn (Y.Y.); gaomeng086630@gmail.com (M.G.); lcy1262@sina.com (C.L.); limingjun@nwsuaf.edu.cn (M.L.); 2 Key Laboratory of Plant Germplasm Enhancement and Specialty Agriculture, Wuhan Botanical Garden of the Chinese Academy of Sciences, Wuhan 430074, China; collee52@gmail.com

**Keywords:** malic acid, citric acid, HPLC, genetic relationship

## Abstract

Significant variation in organic acid components was detected in mature fruits of 101 apple accessions using high-performance liquid chromatography. The *Malus* species predominantly accumulated malic acid and citric acid, whereas wild fruits exhibited significantly higher levels of organic acid content than that in cultivated fruits. Differential accumulation patterns during fruit developmental stages was detected between malic acid and citric acid, thus suggesting a complex genetic regulation mechanism of organic acid metabolism in apple fruit. A highly positive correlation was detected between fruit total organic acid content with malic acid and citric acid content, thus suggesting that malic acid and citric acid are the principal determinants of apple fruit acidity. In contrast to malic acid, citric acid was predominantly detected in partial wild apples, while extremely low to undetectable concentrations of citric acid were observed in cultivated apple fruits; this is likely due to the genetic effects of parental characters. Our results provide vital information that could be useful for future studies on genetic analysis and improvement of organic acid accumulation in apple fruits.

## 1. Introduction

Fruit taste is an important trait majorly controlled by organic acids and, together with aromatic volatile compounds and soluble sugars, they strongly facilitate overall organoleptic quality and fruit stability [1,2]. Three major organic acids that accumulate in most fruits include malic, citric, and tartaric acid, and their final concentration in ripening fruits depends on the balance between the biosynthesis of organic acid, their degradation, and their vacuolar storage [3,4]. Organic acids are different in various kinds of fruits. For example, citric acid is the major organic acid in citrus [5], while malic acid is the predominant organic acid in apple [6], pear [7], and loquat [2]. In addition, organic acids are the main soluble constituents which influence important fruit qualities such as fruit coloration, the shelf life of fresh fruits, and ripeness; consequently, they can be used as an index of consumer acceptability [8]. Moreover, organic acids are also widely used as preservatives, antioxidants, acidulants, and drug absorption modifiers [9].

Apple (*Malus domestica* Borkh.) is a major fruit crop in temperate zones. Market preferences, such as good taste, high nutritional properties, storability, and convenience, caused dessert apples to be increasingly popular. To date, high-performance liquid chromatography (HPLC) and liquid chromatography with tandem-mass spectrometry were widely used for the detection and determination of organic acids in many different fruits and vegetables, such as berry species [8], peach and nectarine [10,11,12], and apple [6,13]. Fruit acidity in cultivated apples is majorly determined by malic acid, which accounts for up to 90% of total organic acids [6]. Citric acid also exists in mature apple fruits; however, it exhibits a very low to undetectable concentration in cultivated apple [14,15]. Previous studies showed that fruit acidity possibly underwent artificial selection during apple domestication [16], and wild *Malus* taxa are considered valuable resources for apple quality improvement. Evidence suggests that, in the course of apple domestication, two species, *M. sieversii* and *M. sylvestris*, acted as the primary and secondary contributors, respectively, to the genome of cultivated varieties [17,18,19,20]. Thus, using wild apple species as natural sources in fruit quality breeding requires an accurate and specific determination of their fruit quality characteristics. However, to our knowledge, other than malic acid, scientific information on organic acid content in *Malus* species is still unknown.

In fruit cells, acidity mainly depends on the metabolism of organic acids, mostly malic acid and citric acid, which can be stored in vacuoles [21,22]. The initial step of cytosolic organic acid (oxaloacetate and malate) synthesis begins with the carboxylation of phosphoenolpyruvate (PEP), which is catalyzed by phosphoenolpyruvate carboxylase (PEPC) and cytosolic nicotinamide adenine dinucleotide (NAD)-dependent malate dehydrogenase (NAD-cytMDH) [23]. The synthesized oxaloacetate and malate in the cytosol are then converted into dicarboxylates and tricarboxylates (citric acid) through the glyoxylate cycle, γ-aminobutyrate shunt, acetyl coenzyme A (acetyl-CoA) catabolism, and the tricarboxylic acid (TCA) cycle [21]. In addition, vacuoles are a major repository for organic acids, which are present at similar concentrations in the cytosol, while their concentrations in the vacuole exceed the corresponding concentrations in the cytosol by several fold [24]. In apple, a gene encoding aluminum-activated malate transporter (ALMT), which is located on tonoplast, is involved in organic acid accumulation [16]. However, the mechanism controlling citric acid metabolism and accumulation in apple fruits remains unclear.

The aim of our work was to understand and to provide an update on the variation of fruit organic acid content in the apple germ plasm by investigating the organic acid components in the mature fruits of 101 apple accessions, including 53 apple cultivars and 58 wild relatives. In addition, we compared variations in organic acid components between apple cultivars and wild relatives and discussed the results. Our study provides new insight into the organic acid evolution during the process of apple domestication, and identifies novel resources which could be useful for future apple breeding.

## 2. Results

### 2.1. HPLC Analysis of Chemical Standards for Organic Acids

**Figure 1:**
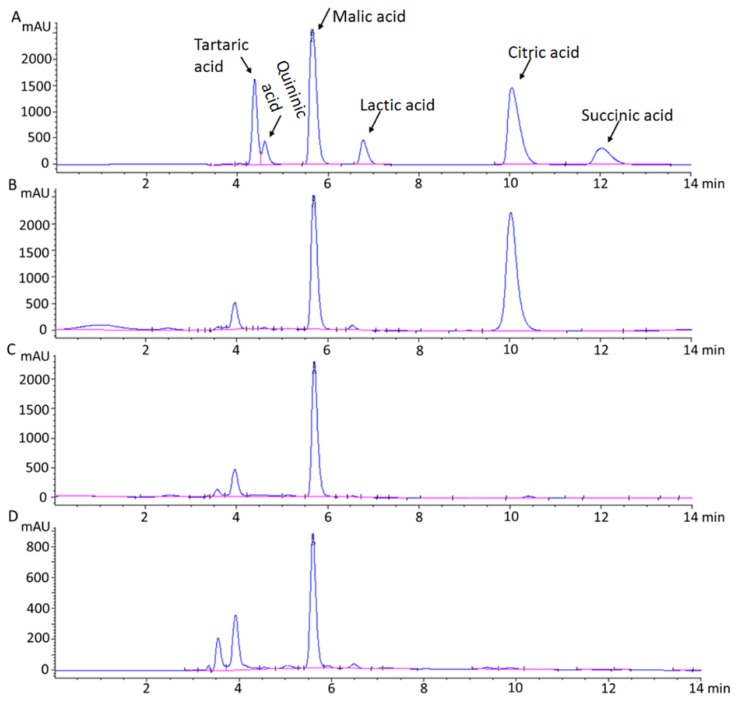
Chromatogram of organic acids obtained at 210 nm: (**A**) standard mixture; (**B**) *Malus toringo*; (**C**) *M. sieversii*; (**D**) *M. sylvestris*. The retention times of tartaric acid, quininic acid, malic acid, lactic acid, citric acid, and succinic acid were 4.371 min, 4.598 min, 5.643 min, 6.766 min, 10.044 min, and 12.034 min, respectively.

To determine the components of organic acids in apple fruits, an artificial mixture containing six acids was prepared according to previous reports [6,10,25], including malic acid, citric acid, tartaric acid, quininic acid, lactic acid, and succinic acid. As shown in Figure 1A, good separation could be achieved in 14 min, and most organic acid components were well separated on the chromatogram. Thus, the chromatographic procedure described above was used to determine the components of organic acids in apple cultivars and wild relatives. Malic acid and citric acid were the two predominant organic acids in *Malus* species, whereas the concentrations of other acids were very low or undetectable (Figure 1).

Based on the assay conditions described, we obtained a linear relationship between the concentration of organic acids (malic acid and citric acid) and peak area at 210 nm. The correlation coefficient of the standards for malic acid and citric acid curves invariably exceeded 0.99. The average regression equations for citric acid and malic acid were *y* = 7967.3*x* + 2634.9 and *y* = 5526.1*x* + 831.3, respectively (Figure S1).

### 2.2. Variation in Organic Acid Components among Malus Species

**Figure 2:**
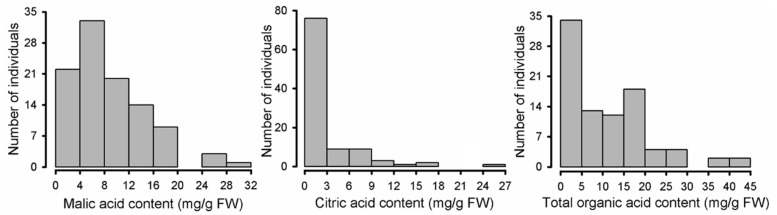
Distribution of organic acids measured for mature fruits of wild and cultivated apple.

The distribution of organic acid characteristics is shown in Figure 2 and Table S1. Malic acid, citric acid, and total organic acid contents exhibited normal distributions with a skew to the right. The total organic acid concentration exhibited significant variation, ranging from 1.72 to 44.63 mg/g fresh weight (FW), with an average of 11.06 mg/g FW (Figure 2; Table S1). Notably, there was almost a 26-fold variation in the total organic acid concentration among the accessions examined. Malic acid was detected in all *Malus* species, ranging from 1.72 to 29.27 mg/g FW, with an average of 8.90 mg/g FW. Citric acid was predominantly detected in several wild apple species, with content ranging from undetectable to 24.24 mg/g FW. The ratios of malic acid to total organic acid and of citric acid to total organic acid were also calculated (Table S1). In cultivated apple fruits, the ratio of malic acid to total organic acid was as high as100%, indicating that malic acid is the predominant organic acid in cultivated apple. Of 58 wild relatives, 31 (53%) revealed a considerable amount of citric acid, with a content of ≥0.43 mg/g FW; the ratio of citric acid to total organic acid ranged from 5.23% to 60.67%, whilst 27 (47%) showed an extremely low to undetectable concentration of citric acid. This indicated that malic acid and citric acid represent the two predominant organic acids in wild apple species.

### 2.3. Dynamic Changes in Malic Acid and Citric Acid Content in Apples at Different Developmental Stages

**Figure 3:**
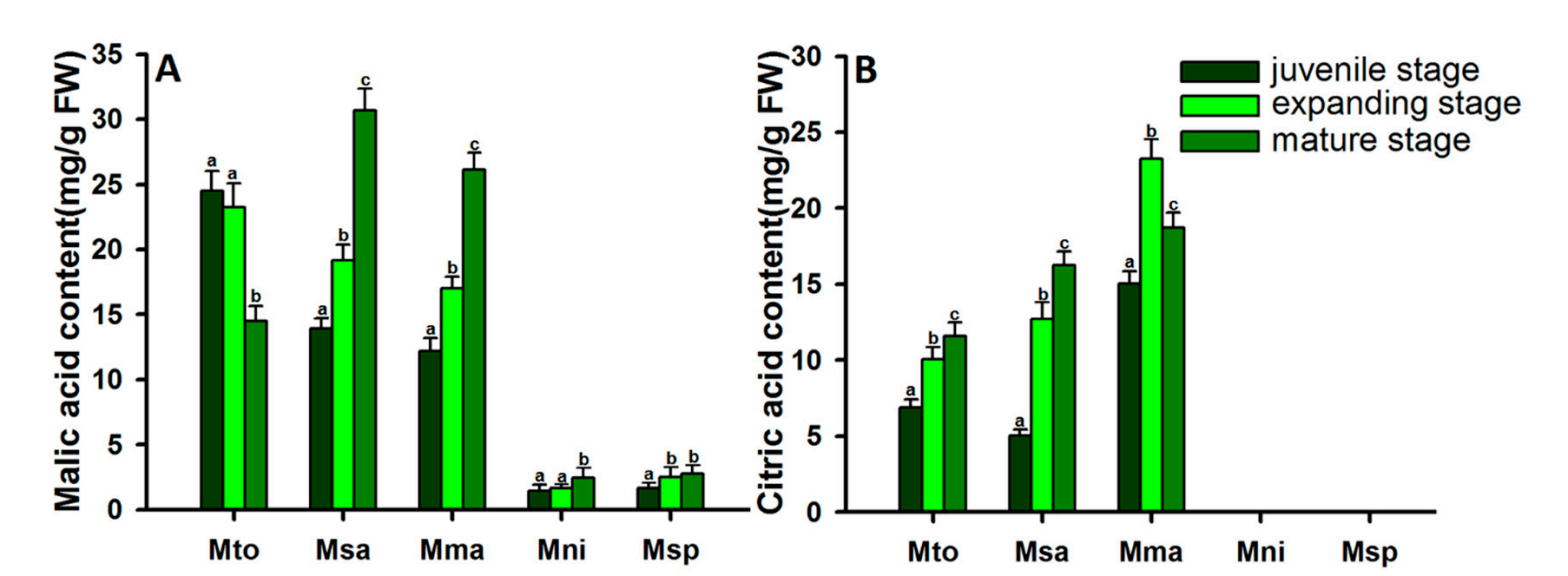
Changes in malic acid and citric acid content in fruits of different apple accessions during fruit development. (**A**): The accumulation pattern of malic acid in fruits of different apple accessions during fruit development. (**B**): The accumulation pattern of citric acid in fruits of different apple accessions during fruit development. Mto, *M. toringoides*; Msa, *M. Sargentii*; Mma, *M. manshurica*; Mni, *M. niedzwetzkyana*; Msp, *M. spectabilis.* Different lowercase letters indicate significant differences of malic acid and citric acid in each apple accession (*p* < 0.05).

A total of five apple accessions were randomly selected for investigation of the variation of malic acid and citric acid content at juvenile, expending, and mature stages (Figure 3). The ripening periods of these five accessions were similar, and maturation occurred at about 90 days after full bloom, showing great variation in malic acid and citric acid content. Of the five accessions, *M. toringoides*, *M. sargentii*, and *M. manshurica* showed high malic acid and citric acid levels, while *M. niedzwetzkyana* and *M. spectabilis* revealed low malic acid content. Furthermore, we also evaluated the accumulation pattern of malic acid and citric acid. Malic acid content was detected in fruits at different developmental stages, but showed different accumulation patterns during fruit development (Figure 3A). For example, during fruit development, increased malic acid content was observed in the accessions *M. sargentii*, *M. manshurica*, *M. niedzwetzkyana*, and *M. spectabilis*, but continuous reduction in malic acid content was detected in the accession *M. toringoides*. Fruit citric acid displayed an increasing accumulation pattern during fruit development in *M. toringoides* and *M. sargentii*; however, it decreased significantly at mature stages in *M. manshurica*. (Figure 3B). The malic acid and citric acid contents showed extreme variation at different developmental stages, suggesting a complex genetic regulation of organic acid metabolism in apples.

### 2.4. Correlation among Organic Acid Contents in Malus Species

Citric acid was only detected in 31 wild apple species; thus, the correlations between citric acid content and malic acid or total organic acid content were calculated using 31 wild apple relatives. The Spearman correlation coefficients are shown in Table 1. Interestingly, a significant positive correlation was detected among organic acid components in mature apple fruits. We detected a highly significant positive correlation between total organic acid and malic acid (*r* = 0.889, *p* < 0.01) and citric acid content (*r* = 0.790, *p* < 0.01), indicating that malic acid and citric acid are predominant and determine fruit acidity. The correlation between malic acid content and citric acid content was also investigated, and the results showed that a significant positive correlation (*r* = 0.357, *p* < 0.05).

### 2.5. Comparison of Organic Acid Contents between Cultivated Apples and Wild Relatives

**Figure 4:**
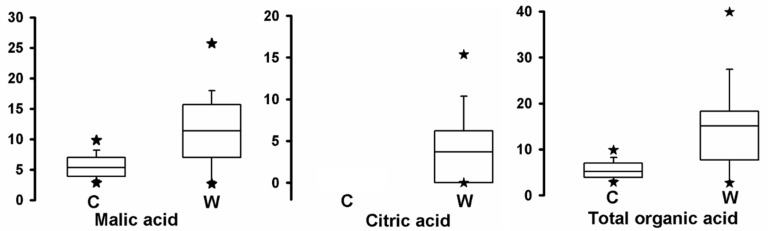
Ranges and distributions of organic acid contents (mg/g fresh weight (FW)) in cultivated and wild apple fruits. The mean values are indicated by horizontal lines in the interior of the box. The distribution for 50% of the data are indicated by the box. Approximately 95% of the data exist within the whiskers. The data that fall outside these whiskers are indicated by solid stars. W, wild fruits; C, cultivated fruits.

The distributions of organic acid contents in wild and cultivated apple fruits are shown in Figure 4 and Table S1. Wild apple fruits exhibited significantly more acid than cultivated fruits, and the average concentration of total organic acid content in wild apple fruits was 2.92 times greater than that in cultivated fruits. This suggests that the variation in fruit acidity is greater in the wild samples than in the cultivated samples. The malic acid content ranged from 2.58 to 29.27 mg/g FW in wild fruits, and from 1.72 to 10.10 mg/g FW in cultivated fruits, and the average concentration of malic acid was significant lower in cultivated fruits (5.26 mg/g FW) than in wild fruits (11.59 mg/g FW), with a statistical value of *p* < 0.001. This indicated that the malic acid concentration also differed significantly between wild and cultivated fruits. In contrast, citric acid concentration was not detected in cultivated apple fruits; however, the citric acid concentration ranged from undetectable to 24.21 mg/g FW in wild apple fruits.

### 2.6. Estimation of Genetic Relationship and Diversity of Organic Acid Components between Apple Cultivars and Wild Relatives

**Figure 5:**
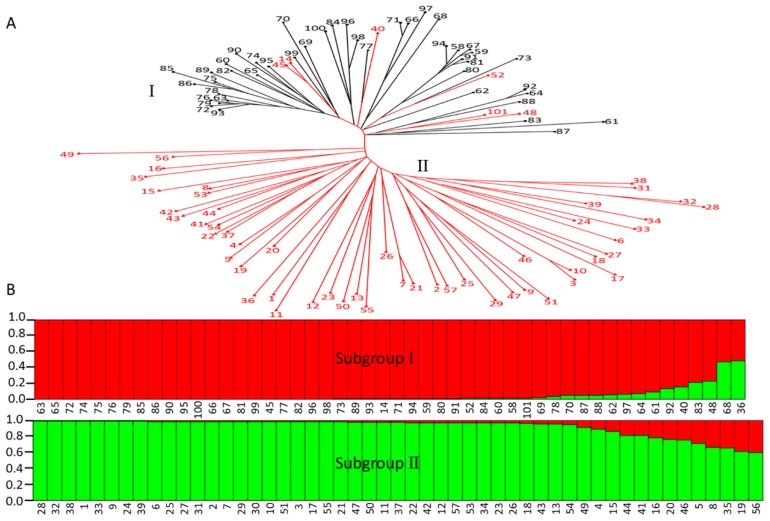
Genetic relationship among 101 apple accessions tested in this study based on 17 simple sequence repeats (SSRs). (**A**) A phylogenetic tree of apple cultivar and wild relatives constructed using SSR data; solid black spots and solid red triangles represent apple cultivars (black line) and wild relatives (red line), respectively. Apple accessions used for the construction of phylogenetic tree are listed in Table S1. (**B**) Bayesian clustering (STRUCTURE *K* = 2) of 101 apple accessions.

In our study, the genetic relationship between apple cultivars and wild relatives was investigated using a total of 17 random simple sequence repeat (SSR) markers (Table 1), and the results showed that all the tested apple accessions were divided into two groups, designated subgroup I and subgroup II (Figure 5A). Subgroup I consisted of 44 apple cultivars and six wild relatives, including two *M. sieversii* accessions, three *M. pumila* accessions, and one *M. sylvestris* accession. Subgroup II was composed of 51 wild apple accessions. Subsequently, population structure analysis was performed to reveal the genetic relationship between apple cultivars and wild relatives, indicating a peak Δ*K* value of *K* = 2 (Figure S2A). This result revealed that there were two distinct subgroups in the tested accessions (Figure 5B), consistent with findings from the phylogenetic tree analysis (Figure 5A). In addition, analysis of organic acid components between different subgroups was also conducted, and the results showed that two predominant organic acid components (malic acid and citric acid) were detected in the apple accessions belonging to subgroup II, and only one predominant organic acid component (malic acid) belonging to subgroup I. *M. sieversii* and *M. sylvestris* were the first and second greatest contributors to the genetic materials of cultivated varieties [16,17,18], and malic acid was the predominant detectable organic acid in their ripening fruits (Figure 1C,D). Thus, our study indicates that the organic acid component difference between apple cultivars and wild relatives is likely to be as a result of direct genetic effects during apple domestication. Moreover, population structure models were further conducted using only wild apple accessions, and three distinct subgroups were identified (Figures S2B and S3). Apple accessions with high citric acid content were evenly distributed among three subgroups, suggesting that the differentiation in organic acid components among *Malus* species occurred prior to the radiation of apple species.

## 3. Discussion

Organic acid is one of the most important components of fruit taste, and impacts on the overall organoleptic quality of apple fruit. In this study, organic acid components were characterized by HPLC, and our results revealed that malic acid and citric acid are the two predominant organic acids in *Malus* species, which is inconsistent with previous findings that malic acid is the major organic acid [6,14,15]. This discrepancy might be explained by the wild apple relatives used in our study. In the present study, our results revealed a dramatic genetic variation for organic acid concentration in the apple germ plasm, and the accumulation patterns of malic acid and citric acid showed great variation at different developmental stages in apple fruits. This is likely due to different genotypes used in this study. In addition, the low level of citric acid is primarily controlled by metabolism of organic acids, and, due to the high concentrations of citrate in cytoplasm, the transport capacity of citrate into the vacuole can be increased [22]. Several studies show that aconitase (ACO) and NAD-dependent isocitrate dehydrogenase (NAD-IDH) control citrate degradation, while citrate synthase (CS) is involved in citrate synthesis [26,27]. Thus, it is worth investigating whether the genes encoding ACO, NAD-IDH, and CS are crucial for the low/high citric acid concentrations in apples. Furthermore, dicarboxylates (malic acid) can be converted into tricarboxylates (citric acid) through the tricarboxylic acid (TCA) cycle, the glyoxylate cycle, γ-aminobutyrate shunt, and acetyl-CoA catabolism, and vice versa [22], which supports the strong correlation between malic and citric acid contents.

It was reported that cultivated apple was domesticated from wild relatives, and various *Malus* species contributed to the genetic makeup of domesticated apple [17,18,19,20,28]. During apple domestication, fruit acidity (or malic acid content) possibly underwent artificial selection [13]. However, the difference in organic acid components cannot be attributed to a selective effect, because citric acid was not detected in cultivated apple fruits. This study revealed two distinct subgroups, with all the apple cultivars and six wild relatives which showed malic acid as the major organic acid clustering into subgroup I (Figure 5; Table S1). During apple domestication, *M. sieversii* and *M. sylvestris* were shown to be the first and second greatest contributors to the genome of cultivated varieties [18,19,20]. Thus, the absence of citric acid in cultivated apple could be considered as the heredity of organic acid components from parental characters. In order to improve this hypothesis, we used an apple segregating F1 population together with parents “Qinguan” and “Honeycrisp” (Figure S4; Table S2). In both the parental “Qinguan” and “Honeycrisp” accessions and the F1 population, malic acid was the predominant detectable organic acid, consistent with the above hypothesis. In addition, 31 wild apple accessions revealed high citric acid content, while 27 wild apple accessions showed low to undetectable citric acid levels, giving an overall 1:1 ratio between wild accessions with varying citric acid content (*x*^2^ = 0.28). Thus, we hypothesize that the citric acid trait in *Malus* species is controlled by a single dominant gene; however, the gene related to citric acid in *Malus* species remains unknown.

## 4. Materials and Methods

### 4.1. Plant Materials

The 101 apple accessions used in this study were maintained at the Horticultural Experimental Station of Northwest A&F University, Yangling, Shaanxi province, China. Of the 101 apple accessions, 53 were apple cultivars while the remaining 58 were wild relatives. Approximately 10 g of young leaves was collected in the spring season, and 100 mg was used for apple genomic DNA extraction.

Leaf samples for 101 apple accessions were immediately frozen in liquid nitrogen, and stored at −80 °C for DNA extraction. Mature fruits were collected in 2016, and the maturity was assessed by checking background color and blush development of the peel, followed by a confirmation of a change in seed color to brown, together with the starch iodine test [29]. Three biological replicates consisting of 10–15 fruits were prepared for each accession. Moreover, five accessions, *M. toringoides*, *M. sargentii*, *M. niedzwetzkyana*, *M. manshurica*, and *M. spectabilis*, were randomly selected for the dynamic variation assay of organic acid accumulation during fruit development. The fruit samples were collected at juvenile, expanding, and mature stages, corresponding to 30, 60, and 90 days after full bloom (DAFB), respectively. All fruit samples were cored and peeled, and the pulps were then cut into small sections, before being immediately frozen in liquid nitrogen, and stored at −70 °C for organic acid measurement by HPLC.

### 4.2. Extraction and Determination of Organic Acids

The measurement of organic acid content was conducted according to a previous protocol with minor modifications [11]. Briefly, each replicate of the fruit samples was ground into a fine powder in liquid nitrogen, and approximately 0.1 g of the powder was dissolved in 1.0 mL of deionized water, obtained from a Milli-Q Element water purification system (Millipore, Bed Ford, MA, USA). The solution was put into an ultrasonic bath for continuous 30-min ultrasonic extraction at room temperature. The solution was then centrifuged at 12,000 rpm (15,294× *g*) for 15 min at 4 °C (5417R centrifuge; Eppendorf, Hamburg, Germany), and the supernatants were filtered through a 0.22-μm Sep-Pak filter (ANPEL, Shanghai, China).

The filtered supernatants were used to measure organic acid content using an Agilent 1260 Infinity HPLC system (Milford, MA, USA). Relative standard curves were used for comparison to accurately determine the concentrations of organic acids in mg/g fresh weight (FW). The injection volume was 20 μL, and organic acids were detected using an HPLC coupled to a diode array detector at 210 nm. An Athena C18 column (100 Å, 4.6 × 250 mm, 5 μm) was used to conduct chromatographic separation, and the column temperature was maintained at 40 °C. The mobile phase was 0.02 mol/L KH_2_PO_4_ solution at pH 2.4, with a flow rate of 0.8 mL/min. All chemical and standards for organic acids were purchased from Sigma (St. Louis, MO, USA), and were dissolved in deionized water.

### 4.3. Data Analysis

All statistical analyses were performed using SPSS statistics 17.0 (SPSS Inc., Chicago, IL, USA). Correlations between experimental variables were examined using Spearman’s rank correlation tests. Independent *t*-tests were performed to evaluate the differences between the means of two groups. Significant differences were estimated using two-tailed tests. Unless otherwise stated, differences were significant at *p* < 0.05. The variation in organic acid content was calculated using SigmaPlot 10.0 (SigmaPlot Software, La Jolla, CA, USA). The dendrogram indicating genetic relationships between apple cultivars and wild relatives was constructed using the DARwin software 6.0 (http://darwin.cirad.fr/darwin/Home.php). Population structure was estimated using the software STRUCTURE 2.0 with the Bayesian model. The parameters were set as follows: burn-in period, 10,000; Markov chain Monte Carlo (MCMC) replication number, 100,000; ten replicate runs for each number of populations (*K*) and *K* from 1 to 10. The optimal *K*-value was calculated based on both ln P(D) in the STRUCTURE output and an ad hoc statistic Δ*K* according to the rate of change in ln P(D) between successive *K*-values [30]. The standard curves for malic acid and citric acid were prepared using a concentration gradient (0.1–10 mg/mL), and, after correction for dilution, the results were expressed in mg/mL.

### 4.4. Genotyping of Apple Germ Plasm

A total of 17 SSR markers distributed across each apple chromosome were randomly chosen to screen the apple germ plasm. Fluorescent dyes (Fam, Hex, and Rox) were labeled at the 5’ end of the forward primer of each SSR marker, and the PCR reaction was conducted in a GeneAmp PCR System 9700 (ABI, Foster City, CA, USA) using the following protocol: initial denaturing for 5 min at 95 °C, followed by 40 cycles consisting of 95 °C for 30 s, 60 °C for 30 s, and 72 °C for 30 s, with a final extension of 72 °C for 10 min. The PCR products were analyzed on an ABI 3730 capillary DNA Genetic Analyzer (ABI, Foster City, CA, USA), and band sizes were estimated by comparing with a GeneScan™ 500 LIZ^®^ Size Standard (ABI, Waltham, MA, USA).

## 5. Conclusions

To our knowledge, this study represents the first large-scale HPLC assessment of organic acid components in wild and cultivated apple fruits. Our results showed that malic acid and citric acid are the two major organic acid components in *Malus* species. In cultivated apple, malic acid is the predominantly detectable organic acid, while malic acid and citric acid are the predominant organic acids in wild apple species. In apple fruits, a highly significant positive correlation of total organic acid content with malic acid and citric acid was detected, while a highly significant positive correlation was also detected between malic acid and citric acid. Wild apple fruits exhibited significantly more acid than cultivated fruits, and showed greater variation in malic acid and citric acid contents than that in cultivated apple fruits. This indicates that wild *Malus* species are useful sources for the genetic improvement of fruit organic acid components in future apple breeding programs. 

Malic acid was detected in all apple cultivars; however, 53% of the wild apple fruits contained high levels of citric acid. In addition, malic acid was the predominant organic acid detected in *M. sieversii* and *M. sylvestris*, which are the first and second greatest contributors to the genetic makeup of cultivated varieties, respectively. Therefore, we attribute the difference in organic acid components between cultivated apple and wild relatives to direct genetic effects, and not selective effects during the process of apple domestication. Moreover, variations in malic acid and citric acid accumulation patterns during developmental stages indicated a complex genetic regulation of organic acid metabolism.

## Figures and Tables

**Table 1:** Simple sequence repeat (SSR) markers used for characterization of apple germ plasm.

SSR	Chr.	Motif	Primer (5’–3’)	
			Forward	Reverse
Hi07d08	1	(GT)_21_	TGACATGCTTTTAGAGGTGGAC	GTTTGAGGGGTGTCCGTACAAG
CH05e03	2	(GA)_26_	CGAATATTTTCACTCTGACTGGG	CAAGTTGTTGTACTGCTCCGAC
Hi15h12	3	(GAT)_6_	GAACAAGAAGGACGCGAATC	GTTTGGGCCTCGTTATCACTACCA
AT000420-SSR	4	(GA)_8_	TTGGACCAATTATCTCTGCTATT	GATGTGGTCAGGGAGAGGAG
Hi11a03	5	(GAA)_8_	GGAATTGGAGCTTGATGCAG	GTTTCATACGGAATGGCAAATCG
CH03c01	6	(CT)_13_	CCTTTTGGCACTAGGCAGAC	CTGCCCTCAAGGAGAATGTC
Hi05b09	7	(AG)_16_	AAACCCAACCCAAAGAGTGG	GTTTCTAACGTGCGCCTAACGTG
Hi04e05	8	(AG)_40_	AAGGGTGTTTGCGGAGTTAG	GGTGCGCTGTCTTCCATAAA
Hi23d06	9	(ACA)_8_	TTGAAACCCGTACATTCAACTC	GTTTCAAGAACCGTGCGAAATG
COL	10	(GA)_17_	AGGAGAAAGGCGTTTACCTG	GACTCATTCTTCGTCGTCACTG
CN491050-SSR	11	(AG)_14_	CGCTGATGCGATAATCAATG	GTTTCACCCACAGAATCACCAGA
CH04d02	12	(TC)_19_	CGTACGCTGCTTCTTTTGCT	CTATCCACCACCCGTCAACT
Hi08e06	13	(TTG)_7_	GCAATGGCGTTCTAGGATTC	GTTTGGCTGCTTGGAGATGTG
CH03g04	14	(GA)_20_	ATGTCCAATGTAGACACGCAAC	TTGAAGATGGCCTAACCTTGTT
Hi15c07	15	(CCA)_6_	TCACTTCCCATCATCACTGC	GTTTCAATGTCGAGGCTGGTAATG
Hi01a08	16	(TCT)_28_	AAGTCCAATCGCACTCACG	CGTAGCTCTCTCCCGATACG
CH01h01	17	(TC)_25_	GAAAGACTTGCAGTGGGAGC	GGAGTGGGTTTGAGAAGGTT

## Supplementary Materials

The following are available online at http://www.mdpi.com/2218-1989/8/4/74/s1: Figure S1: The standard curves of citric acid and malic acid were prepared using different concentrations. Figure S2: Determination of the optimal value of K and inferred population structure of the apple population. The dot represents the ad hoc procedure described in Pritchard et al. (2000), and the star indicates the second order of statistics (Delta K) based on Evanno et al. (2005). A and B show the determination of the optimal value of K and inferred population structure of all the apple accession and wild relatives tested in in this study, respectively. Figure S3: Bayesian clustering (STRUCTURE K = 3) of 58 apple wild relatives. Red numbers represent apple accessions which contained high citric acid concentration. Figure S4: Chromatogram of organic acids of ‘Qinguan’ x ‘Honeycrysp’ population and a sample solution obtained from fresh apple fruits at 210 nm. A: Qingguan; B: Honeycrisp; C: 4-113, a hybrid offspring from ‘Qinguan’ x ‘Honeycrysp’. Table S1: Organic acid content of 101 apple accessions used in this study. Table S2: Organic acid content of ‘Qinguan’ x ‘Honeycrysp’ population.
