# Supplementary material for: Determination of Predominant Organic Acid Components in Malus Species: Correlation with Apple Domestication
Source: Metabolites. 2018 Oct 31;8(4):74. doi: 10.3390/metabo8040074 (PMC6316603; doi:10.3390/metabo8040074)
Supplement: Supplementary file 1 [file metabolites-08-00074-s001.zip › Supplimentary/supplimental tables.docx]

Table S1. Organic acid content of 101 apple accessions used in this study.

| Number | Species | MA | CA | TOA | MA/TOA(%) | CA/TOA(%) |
| --- | --- | --- | --- | --- | --- | --- |
| 1 | *Malus honanensis* | 5.64 | 0.00 | 5.64 | 100.00 | 0.00 |
| 2 | *Malus micromalus* | 9.34 | 5.43 | 14.76 | 63.28 | 36.72 |
| 3 | *Malus robusta* | 12.50 | 5.68 | 18.18 | 68.76 | 31.24 |
| 4 | *Malus prunifolia* | 7.77 | 0.43 | 8.20 | 97.76 | 5.24 |
| 5 | *Malus prunifolia* | 4.18 | 3.84 | 8.03 | 52.05 | 47.95 |
| 6 | *Malus prunifolia* | 17.58 | 5.83 | 23.41 | 75.10 | 24.90 |
| 7 | *Malus prunifolia* | 8.15 | 7.23 | 15.38 | 52.99 | 47.01 |
| 8 | *Malus sieversii* | 2.79 | 0.00 | 2.79 | 100.00 | 0.00 |
| 9 | *Malus prunifolia* | 10.80 | 6.49 | 17.29 | 62.46 | 37.54 |
| 10 | *Malus robusta* | 13.28 | 5.02 | 18.30 | 72.57 | 27.43 |
| 11 | *Malus* *ombrophila* | 19.93 | 0.00 | 19.93 | 100.00 | 0.00 |
| 12 | *Malus rockii* | 15.69 | 24.21 | 39.90 | 39.32 | 60.68 |
| 13 | *Malus toringoides* | 14.94 | 11.16 | 26.10 | 57.24 | 42.76 |
| 14 | *Malus sieversii* | 10.57 | 0.00 | 10.57 | 100.00 | 0.00 |
| 15 | *Malus halliana* | 3.72 | 0.00 | 3.72 | 100.00 | 0.00 |
| 16 | *Malus yunnaensis* | 10.27 | 6.64 | 16.91 | 60.73 | 39.27 |
| 17 | *Malus sargentii* | 29.27 | 15.36 | 44.63 | 65.58 | 34.42 |
| 18 | *Malus sp.* | 17.22 | 10.39 | 27.61 | 62.37 | 37.63 |
| 19 | *Malus halliana* | 12.68 | 3.63 | 16.31 | 77.74 | 22.23 |
| 20 | *Malus halliana* | 16.39 | 6.13 | 22.52 | 72.78 | 27.22 |
| 21 | *Malus prunifolia* | 10.04 | 3.72 | 13.76 | 72.97 | 27.03 |
| 22 | *Malus niedzwetzkyana* | 10.45 | 0.00 | 10.45 | 100.00 | 0.00 |
| 23 | *Malus asatica* | 7.10 | 0.00 | 7.10 | 100.00 | 0.00 |
| 24 | *Malus hybrid* | 12.56 | 0.00 | 12.56 | 100.00 | 0.00 |
| 25 | *Malus hybrid* | 14.13 | 2.19 | 16.32 | 86.58 | 13.42 |
| 26 | *Malus adstringens* | 10.19 | 0.00 | 10.19 | 100.00 | 0.00 |
| 27 | *Malus hybrid* | 25.71 | 12.68 | 38.39 | 66.97 | 33.03 |
| 28 | *Malus purpurea* | 13.49 | 0.00 | 13.49 | 100.00 | 0.00 |
| 29 | *Malus hybrid* | 5.94 | 6.23 | 12.17 | 48.81 | 51.19 |
| 30 | *Malus hybrid* | 8.63 | 7.53 | 16.16 | 53.40 | 46.60 |
| 31 | *Malus sp.* | 15.83 | 2.48 | 18.31 | 86.46 | 13.54 |
| 32 | *Malus hybrid* | 15.23 | 0.00 | 15.23 | 100.00 | 0.00 |
| 33 | *Malus hybrid* | 17.13 | 10.31 | 27.44 | 62.43 | 37.57 |
| 34 | *Malus hybrid* | 10.75 | 5.49 | 16.24 | 66.19 | 33.81 |
| 35 | *Malus hybrid* | 15.83 | 2.34 | 18.17 | 87.12 | 12.88 |
| 36 | *Malus sp.* | 16.53 | 8.01 | 24.54 | 67.36 | 32.64 |
| 37 | *Malus niedzwetzkyana* | 2.68 | 0.00 | 2.68 | 100.00 | 0.00 |
| 38 | *Malus hybrid* | 6.99 | 0.00 | 6.99 | 100.00 | 0.00 |
| 39 | *Malus hybrid* | 24.06 | 2.98 | 27.04 | 88.98 | 11.02 |
| 40 | *Malus sieversii* | 10.76 | 0.00 | 10.76 | 100.00 | 0.00 |
| 41 | *Malus sieversii* | 2.76 | 0.00 | 2.76 | 100.00 | 0.00 |
| 42 | *Malus soulardii* | 10.65 | 0.98 | 11.63 | 91.57 | 8.43 |
| 43 | *Malus sieversii* | 17.94 | 0.00 | 17.94 | 100.00 | 0.00 |
| 44 | *Malus sieversii* | 9.97 | 0.00 | 9.97 | 100.00 | 0.00 |
| 45 | *Malus pumila* | 3.42 | 0.00 | 3.42 | 100.00 | 0.00 |
| 46 | *Malus sp.* | 15.03 | 0.00 | 15.03 | 100.00 | 0.00 |
| 47 | *Malus sp.* | 13.63 | 3.42 | 17.05 | 79.94 | 20.06 |
| 48 | *Malus pumila* | 8.42 | 0.00 | 8.42 | 100.00 | 0.00 |
| 49 | *Malus asiatica* | 7.71 | 0.00 | 7.71 | 100.00 | 0.00 |
| 50 | *Malus niedzwetzkyana* | 17.34 | 0.00 | 17.34 | 100.00 | 0.00 |
| 51 | *Malus pumila* | 11.57 | 6.86 | 18.43 | 62.78 | 37.22 |
| 52 | *Malus pumila* | 2.58 | 0.00 | 2.58 | 100.00 | 0.00 |
| 53 | *Malus sieversii* | 3.08 | 0.00 | 3.08 | 100.00 | 0.00 |
| 54 | *Malus spectabilis* | 2.61 | 0.00 | 2.61 | 100.00 | 0.00 |
| 55 | *Malus spectabilis* | 7.00 | 0.00 | 7.00 | 100.00 | 0.00 |
| 56 | *Malus bhutanica* | 12.17 | 8.18 | 20.35 | 59.80 | 40.20 |
| 57 | *Malus manshurica* | 26.14 | 17.92 | 44.06 | 59.33 | 40.67 |
| 58 | *Malus domestica* | 4.08 | 0.00 | 4.08 | 100.00 | 0.00 |
| 59 | *Malus domestica* | 3.78 | 0.00 | 3.78 | 100.00 | 0.00 |
| 60 | *Malus domestica* | 8.00 | 0.00 | 8.00 | 100.00 | 0.00 |
| 61 | *Malus domestica* | 10.10 | 0.00 | 10.10 | 100.00 | 0.00 |
| 62 | *Malus domestica* | 6.92 | 0.00 | 6.92 | 100.00 | 0.00 |
| 63 | *Malus domestica* | 3.62 | 0.00 | 3.62 | 100.00 | 0.00 |
| 64 | *Malus domestica* | 7.34 | 0.00 | 7.34 | 100.00 | 0.00 |
| 65 | *Malus domestica* | 5.62 | 0.00 | 5.62 | 100.00 | 0.00 |
| 66 | *Malus domestica* | 6.62 | 0.00 | 6.62 | 100.00 | 0.00 |
| 67 | *Malus domestica* | 3.66 | 0.00 | 3.66 | 100.00 | 0.00 |
| 68 | *Malus domestica* | 10.03 | 0.00 | 10.03 | 100.00 | 0.00 |
| 69 | *Malus domestica* | 3.19 | 0.00 | 3.19 | 100.00 | 0.00 |
| 70 | *Malus domestica* | 5.22 | 0.00 | 5.22 | 100.00 | 0.00 |
| 71 | *Malus domestica* | 5.55 | 0.00 | 5.55 | 100.00 | 0.00 |
| 72 | *Malus domestica* | 2.79 | 0.00 | 2.79 | 100.00 | 0.00 |
| 73 | *Malus domestica* | 8.62 | 0.00 | 8.62 | 100.00 | 0.00 |
| 74 | *Malus domestica* | 7.35 | 0.00 | 7.35 | 100.00 | 0.00 |
| 75 | *Malus domestica* | 5.65 | 0.00 | 5.65 | 100.00 | 0.00 |
| 76 | *Malus domestica* | 3.37 | 0.00 | 3.37 | 100.00 | 0.00 |
| 77 | *Malus domestica* | 3.90 | 0.00 | 3.90 | 100.00 | 0.00 |
| 78 | *Malus domestica* | 4.91 | 0.00 | 4.91 | 100.00 | 0.00 |
| 79 | *Malus domestica* | 4.13 | 0.00 | 4.13 | 100.00 | 0.00 |
| 80 | *Malus domestica* | 4.58 | 0.00 | 4.58 | 100.00 | 0.00 |
| 81 | *Malus domestica* | 4.01 | 0.00 | 4.01 | 100.00 | 0.00 |
| 82 | *Malus domestica* | 8.33 | 0.00 | 8.33 | 100.00 | 0.00 |
| 83 | *Malus domestica* | 1.72 | 0.00 | 1.72 | 100.00 | 0.00 |
| 84 | *Malus domestica* | 7.54 | 0.00 | 7.54 | 100.00 | 0.00 |
| 85 | *Malus domestica* | 4.24 | 0.00 | 4.24 | 100.00 | 0.00 |
| 86 | *Malus domestica* | 4.64 | 0.00 | 4.64 | 100.00 | 0.00 |
| 87 | *Malus domestica* | 4.73 | 0.00 | 4.73 | 100.00 | 0.00 |
| 88 | *Malus domestica* | 4.21 | 0.00 | 4.21 | 100.00 | 0.00 |
| 89 | *Malus domestica* | 4.84 | 0.00 | 4.84 | 100.00 | 0.00 |
| 90 | *Malus domestica* | 7.17 | 0.00 | 7.17 | 100.00 | 0.00 |
| 91 | *Malus domestica* | 7.60 | 0.00 | 7.60 | 100.00 | 0.00 |
| 92 | *Malus domestica* | 5.81 | 0.00 | 5.81 | 100.00 | 0.00 |
| 93 | *Malus domestica* | 4.03 | 0.00 | 4.03 | 100.00 | 0.00 |
| 94 | *Malus domestica* | 3.91 | 0.00 | 3.91 | 100.00 | 0.00 |
| 95 | *Malus domestica* | 3.94 | 0.00 | 3.94 | 100.00 | 0.00 |
| 96 | *Malus domestica* | 3.50 | 0.00 | 3.50 | 100.00 | 0.00 |
| 97 | *Malus domestica* | 5.95 | 0.00 | 5.95 | 100.00 | 0.00 |
| 98 | *Malus domestica* | 3.20 | 0.00 | 3.20 | 100.00 | 0.00 |
| 99 | *Malus domestica* | 4.30 | 0.00 | 4.30 | 100.00 | 0.00 |
| 100 | *Malus domestica* | 3.18 | 0.00 | 3.18 | 100.00 | 0.00 |
| 101 | *Malus sylvestris* | 3.81 | 0.00 | 3.81 | 100.00 | 0.00 |

MA: malic acid content; CA: citric acid content; TOA: total organic acid content (mg/g FW).

Table S2. Organic acid content of ‘Qinguan’ x ‘Honeycrysp’ population

| Number | Name | MA | CA | TOA |
| --- | --- | --- | --- | --- |
| 1 | Honeycrysp | 6.05 | 0.00 | 6.05 |
| 2 | Qingguan | 1.68 | 0.00 | 1.68 |
| 3 | 4-40 | 5.39 | 0.00 | 5.39 |
| 4 | 3-114 | 6.43 | 0.00 | 6.43 |
| 5 | 4-109 | 6.88 | 0.00 | 6.88 |
| 6 | 4-74 | 6.02 | 0.00 | 6.02 |
| 7 | 4-72 | 3.72 | 0.00 | 3.72 |
| 8 | 3-38 | 5.84 | 0.00 | 5.84 |
| 9 | 4-45 | 3.78 | 0.00 | 3.78 |
| 10 | 4-89 | 3.04 | 0.00 | 3.04 |
| 11 | 4-32 | 6.14 | 0.00 | 6.14 |
| 12 | 4-123 | 6.49 | 0.00 | 6.49 |
| 13 | 5-126 | 8.93 | 0.00 | 8.93 |
| 14 | 3-76 | 4.65 | 0.00 | 4.65 |
| 15 | 5-115 | 7.25 | 0.00 | 7.25 |
| 16 | 5-135 | 3.87 | 0.00 | 3.87 |
| 17 | 5-56 | 7.11 | 0.00 | 7.11 |
| 18 | 4-120 | 3.30 | 0.00 | 3.30 |
| 19 | 4-78 | 9.50 | 0.00 | 9.50 |
| 20 | 5-35 | 3.98 | 0.00 | 3.98 |
| 21 | 5-65 | 6.28 | 0.00 | 6.28 |
| 22 | 4-119 | 8.10 | 0.00 | 8.10 |
| 23 | 5-83 | 6.76 | 0.00 | 6.76 |
| 24 | 4-57 | 6.49 | 0.00 | 6.49 |
| 25 | 3-14 | 2.39 | 0.00 | 2.39 |
| 26 | 3-75 | 4.36 | 0.00 | 4.36 |
| 27 | 3-39 | 5.00 | 0.00 | 5.00 |
| 28 | 3-50 | 6.22 | 0.00 | 6.22 |
| 29 | 4-88 | 4.67 | 0.00 | 4.67 |
| 30 | 5-86 | 4.26 | 0.00 | 4.26 |
| 31 | 4-83 | 7.80 | 0.00 | 7.80 |
| 32 | 4-116 | 6.32 | 0.00 | 6.32 |
| 33 | 4-102 | 0.56 | 0.00 | 0.56 |
| 34 | 5-70 | 4.19 | 0.00 | 4.19 |
| 35 | 4-122 | 7.87 | 0.00 | 7.87 |
| 36 | 5-106 | 6.86 | 0.00 | 6.86 |
| 37 | 4-94 | 4.84 | 0.00 | 4.84 |
| 38 | 4-51 | 6.22 | 0.00 | 6.22 |
| 39 | 3-131 | 6.14 | 0.00 | 6.14 |
| 40 | 3-8 | 4.37 | 0.00 | 4.37 |
| 41 | 3-131 | 6.03 | 0.00 | 6.03 |
| 42 | 3-11 | 8.28 | 0.00 | 8.28 |
| 43 | 4-158 | 8.65 | 0.00 | 8.65 |
| 44 | 3-127 | 5.25 | 0.00 | 5.25 |
| 45 | 3-147 | 7.82 | 0.00 | 7.82 |
| 46 | 5-80 | 5.64 | 0.00 | 5.64 |
| 47 | 4-155 | 2.35 | 0.00 | 2.35 |
| 48 | 4-158 | 7.93 | 0.00 | 7.93 |
| 49 | 5-118 | 5.85 | 0.00 | 5.85 |
| 50 | 3-72 | 6.87 | 0.00 | 6.87 |
| 51 | 3-37 | 10.46 | 0.00 | 10.46 |
| 52 | 3-22 | 3.91 | 0.00 | 3.91 |
| 53 | 4-34 | 5.81 | 0.00 | 5.81 |
| 54 | 4-71 | 5.42 | 0.00 | 5.42 |
| 55 | 5-92 | 5.58 | 0.00 | 5.58 |
| 56 | 3-10 | 12.90 | 0.00 | 12.90 |
| 57 | 5-125 | 4.83 | 0.00 | 4.83 |
| 58 | 5-63 | 4.06 | 0.00 | 4.06 |
| 59 | 3-150 | 7.14 | 0.00 | 7.14 |
| 60 | 3-74 | 11.14 | 0.00 | 11.14 |
| 61 | 4-13 | 4.22 | 0.00 | 4.22 |
| 62 | 4-117 | 4.95 | 0.00 | 4.95 |
| 63 | 5-156 | 5.36 | 0.00 | 5.36 |
| 64 | 5-77 | 3.85 | 0.00 | 3.85 |
| 65 | 3-24 | 5.84 | 0.00 | 5.84 |
| 66 | 3-126 | 2.86 | 0.00 | 2.86 |
| 67 | 3-28 | 7.31 | 0.00 | 7.31 |
| 68 | 5-89 | 6.55 | 0.00 | 6.55 |
| 69 | 4-105 | 4.24 | 0.00 | 4.24 |
| 70 | 4-126 | 2.72 | 0.00 | 2.72 |
| 71 | 4-149 | 6.45 | 0.00 | 6.45 |
| 72 | 5-82 | 3.15 | 0.00 | 3.15 |
| 73 | 4-85 | 4.55 | 0.00 | 4.55 |
| 74 | 5-26 | 7.74 | 0.00 | 7.74 |
| 75 | 3-146 | 3.56 | 0.00 | 3.56 |
| 76 | 4-113 | 6.01 | 0.00 | 6.01 |
| 77 | 4-148 | 8.98 | 0.00 | 8.98 |
| 78 | 3-125 | 4.89 | 0.00 | 4.89 |
| 79 | 3-101 | 5.97 | 0.00 | 5.97 |
| 80 | 5-75 | 4.17 | 0.00 | 4.17 |
| 81 | 3-57 | 8.53 | 0.00 | 8.53 |
| 82 | 6-10 | 3.43 | 0.00 | 3.43 |
| 83 | 3-137 | 4.77 | 0.00 | 4.77 |
| 84 | 3-124 | 4.30 | 0.00 | 4.30 |
| 85 | 3-26 | 6.41 | 0.00 | 6.41 |
| 86 | 4-159 | 5.67 | 0.00 | 5.67 |
| 87 | 3-5 | 5.60 | 0.00 | 5.60 |
| 88 | 6-6 | 7.85 | 0.00 | 7.85 |
| 89 | 5-120 | 3.82 | 0.00 | 3.82 |
| 90 | 3-21 | 3.84 | 0.00 | 3.84 |
| 91 | 3-101 | 5.85 | 0.00 | 5.85 |
| 92 | 4-101 | 6.48 | 0.00 | 6.48 |
| 93 | 3-59 | 4.71 | 0.00 | 4.71 |
| 94 | 6-15 | 5.82 | 0.00 | 5.82 |
| 95 | 3-34 | 4.59 | 0.00 | 4.59 |
| 96 | 3-151 | 5.03 | 0.00 | 5.03 |
| 97 | 5-121 | 3.93 | 0.00 | 3.93 |
| 98 | 5-120 | 3.76 | 0.00 | 3.76 |
| 99 | 3-153 | 5.84 | 0.00 | 5.84 |
| 100 | 5-21 | 4.09 | 0.00 | 4.09 |
| 101 | 3-33 | 3.97 | 0.00 | 3.97 |
| 102 | 4-31 | 4.44 | 0.00 | 4.44 |
| 103 | 3-77 | 7.60 | 0.00 | 7.60 |
| 104 | 3-34 | 4.18 | 0.00 | 4.18 |
| 105 | 3-58 | 3.68 | 0.00 | 3.68 |
| 106 | 5-100 | 4.55 | 0.00 | 4.55 |
| 107 | 5-46 | 3.45 | 0.00 | 3.45 |
| 108 | 3-70 | 3.68 | 0.00 | 3.68 |
| 109 | 3-135 | 6.13 | 0.00 | 6.13 |
| 110 | 3-21 | 4.38 | 0.00 | 4.38 |
| 111 | 3-107 | 5.91 | 0.00 | 5.91 |
| 112 | 3-96 | 3.94 | 0.00 | 3.94 |
| 113 | 3-81 | 5.95 | 0.00 | 5.95 |
| 114 | 3-102 | 5.88 | 0.00 | 5.88 |
| 115 | 4-8 | 5.94 | 0.00 | 5.94 |
| 116 | 3-61 | 4.30 | 0.00 | 4.30 |
| 117 | 3-65 | 5.77 | 0.00 | 5.77 |
| 118 | 3-73 | 4.42 | 0.00 | 4.42 |
| 119 | 3-9 | 5.20 | 0.00 | 5.20 |
| 120 | 3-53 | 6.02 | 0.00 | 6.02 |
| 121 | 5-9 | 6.76 | 0.00 | 6.76 |
| 122 | 3-54 | 5.04 | 0.00 | 5.04 |
| 123 | 3-91 | 4.26 | 0.00 | 4.26 |
| 124 | 3-43 | 3.88 | 0.00 | 3.88 |
| 125 | 3-87 | 5.17 | 0.00 | 5.17 |
| 126 | 3-123 | 4.07 | 0.00 | 4.07 |
| 127 | 4-2 | 5.33 | 0.00 | 5.33 |
| 128 | 4-26 | 5.36 | 0.00 | 5.36 |
| 129 | 4-139 | 7.20 | 0.00 | 7.20 |
| 130 | 3-55 | 4.00 | 0.00 | 4.00 |
| 131 | 3-50 | 5.05 | 0.00 | 5.05 |
| 132 | 3-152 | 6.98 | 0.00 | 6.98 |

MA: malic acid content; CA: citric acid content; TOA: total organic acid content (mg/g FW).
