# Supplementary figures and images for: Determination of Predominant Organic Acid Components in Malus Species: Correlation with Apple Domestication
Source: Metabolites. 2018 Oct 31;8(4):74. doi: 10.3390/metabo8040074 (PMC6316603; doi:10.3390/metabo8040074)

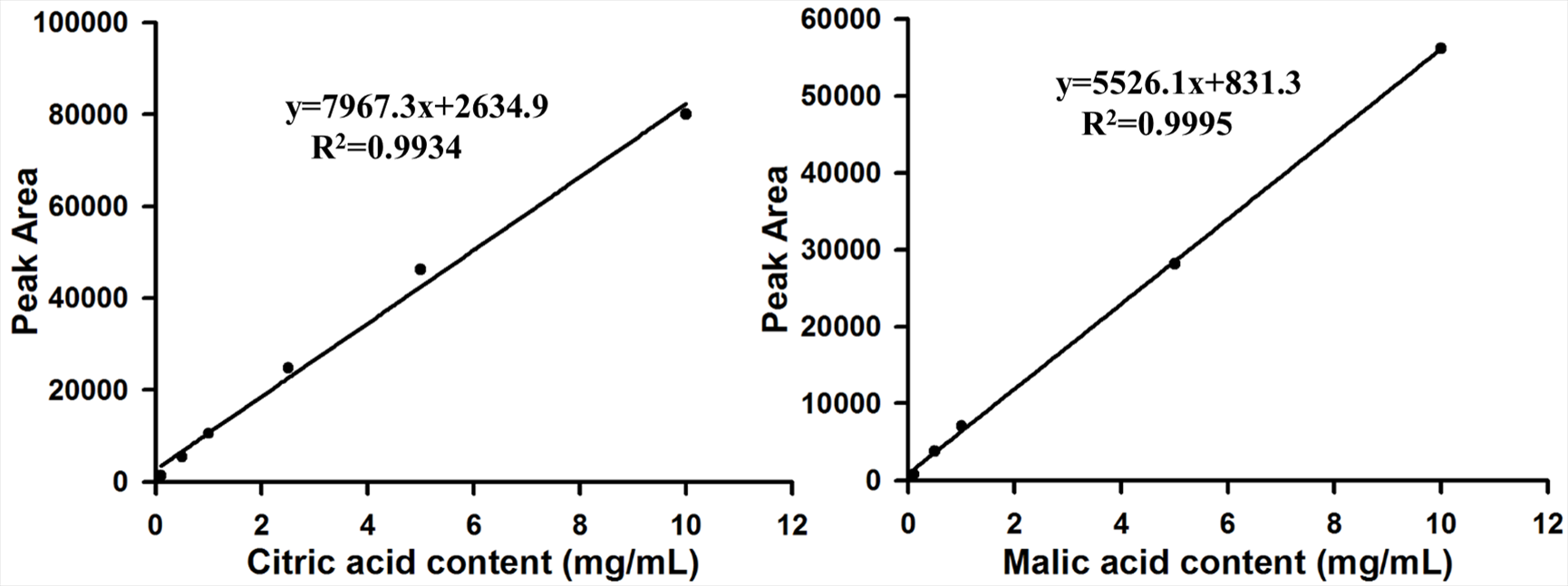

Supplement: Supplementary file 1 [file metabolites-08-00074-s001.zip › Supplimentary/Fig.S1.TIF]

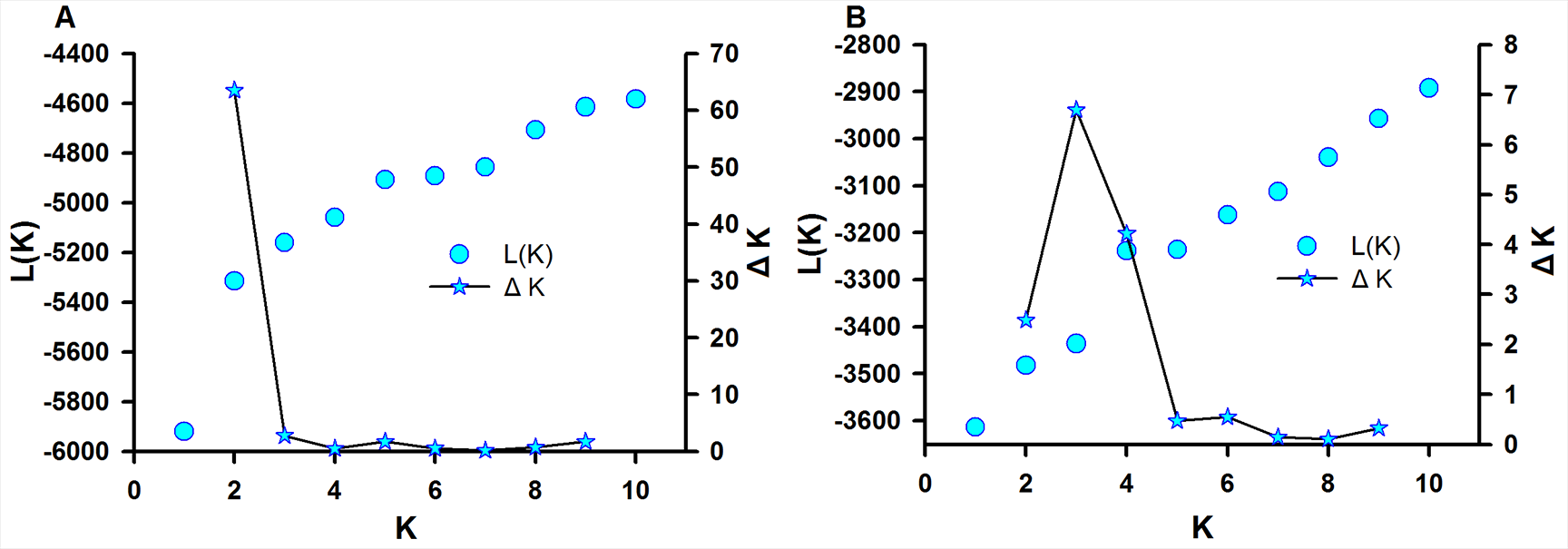

Supplement: Supplementary file 1 [file metabolites-08-00074-s001.zip › Supplimentary/Fig.S2.TIF]

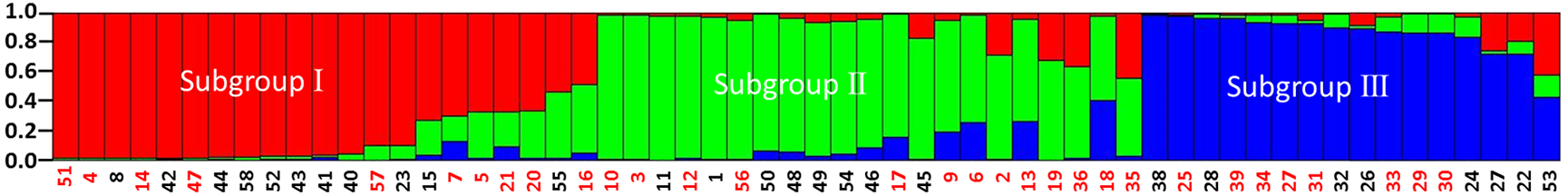

Supplement: Supplementary file 1 [file metabolites-08-00074-s001.zip › Supplimentary/Fig.S3.tif]

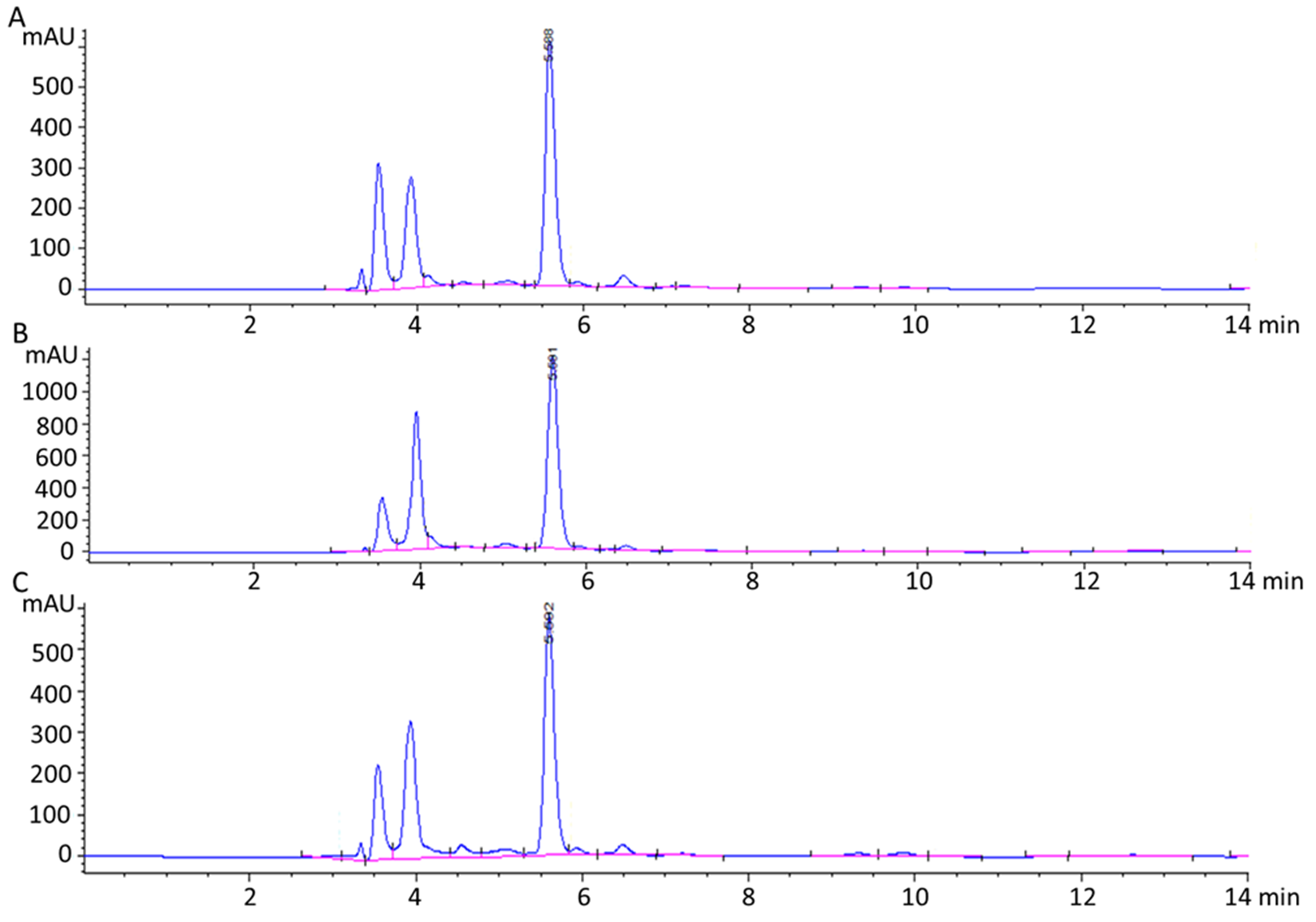

Supplement: Supplementary file 1 [file metabolites-08-00074-s001.zip › Supplimentary/Fig.S4.tif]
